# Ophthalmic artery occlusion following local hyaluronic acid injection: A case report

**DOI:** 10.1097/MD.0000000000050489

**Published:** 2026-09-11

**Authors:** Ziqi Xiong, Haiming Lv, Chunhuan Niu, Ying Wen, Yane Gao

**Affiliations:** a Ophthalmology and Optometry Medical School, Shandong University of Traditional Chinese Medicine, Jinan, Shandong Province, China; b Affiliated Eye Hospital of Shandong University of Traditional Chinese Medicine, Shandong Academy of Eye Disease Prevention and Therapy, Shandong Provincial Key Laboratory of Integrated Traditional Chinese and Western Medicine for Prevention and Therapy of Ocular Diseases, Jinan, Shandong Province, China.

**Keywords:** complications, facial filler, hyaluronic acid, ophthalmic artery occlusion

## Abstract

**Rationale:**

Ophthalmic artery occlusion (OAO) following hyaluronic acid (HA) injection is a rare but devastating complication of facial aesthetic procedures, often resulting in profound and irreversible visual loss. Due to its rarity and poor prognosis, standardized management of iatrogenic OAO remains limited. To our knowledge, long-term multimodal imaging follow-up of HA-induced OAO with integrated traditional Chinese and Western medicine treatment is rarely documented. Here, we report a case of OAO following nasal root HA injection and describe its clinical manifestations, multimodal imaging findings, and 10-month follow-up outcomes.

**Patient concerns:**

A 46-year-old female initially presented with sudden vision loss (no light perception) in the left eye and skin mottling after HA injection into the nasal bridge. After receiving intravenous dexamethasone and oxygen inhalation at a local hospital, she was transferred for interventional thrombolysis twice. However, no significant visual improvement was observed, and the patient was subsequently referred to our hospital for further diagnosis and treatment.

**Diagnoses:**

A definitive diagnosis of ophthalmic artery occlusion was not initially established at the first visit, where the patient was diagnosed with central retinal artery occlusion of the left eye. Upon presentation to our hospital, the left eye showed no light perception, intraocular pressure of 8 mm Hg, marked anterior segment inflammation, diffuse retinal edema, and a pale optic disc. Optical coherence tomography, flash visual evoked potential, electroretinography, and B-scan further confirmed the findings. Thus, the patient was diagnosed with left ophthalmic artery occlusion following HA injection.

**Interventions:**

Integrated traditional Chinese and Western medicine treatment, including anti-ischemic therapy, neuroprotection, herbal decoction, and acupuncture, was administered to the left eye.

**Outcomes:**

The visual acuity of the left eye remained no light perception throughout the follow-up. Scanning laser ophthalmoscopy demonstrated progressive retinal atrophy and proliferative membrane formation during the 9-month follow-up period.

**Lessons:**

Although iatrogenic ophthalmic artery occlusion following HA injection is rare, ophthalmologists should remain vigilant due to its poor prognosis and narrow therapeutic window. Standardized injection techniques and rigorous risk prevention measures should be prioritized to optimize patient safety.

## 1. Introduction

Hyaluronic acid (HA) fillers are widely used in aesthetic medicine because of their favorable safety profile and immediate cosmetic effects. However, rare but severe vascular complications may occur, including ophthalmic artery occlusion (OAO), retinal artery occlusion, cerebral infarction, and permanent blindness.^[1-3]^ Among these, OAO is one of the most devastating complications, often resulting in profound and irreversible visual loss.^[1,4]^

OAO is usually caused by inadvertent intravascular injection and embolization of filler material through anastomotic channels between the facial and ophthalmic arterial systems.^[2-4]^ Because of its rapid progression, limited therapeutic window, and poor visual prognosis, early recognition and prevention are crucial.^[5,6]^ Herein, we report a case of OAO following HA injection into the nasal root and describe its clinical manifestations, multimodal imaging findings, treatment, and long-term follow-up outcomes.

## 2. Case presentation

The patient was a 46-year-old woman who sought treatment at a local ophthalmic hospital for sudden loss of vision in the left eye, following a HA injection at the nasal root. She was diagnosed with “central retinal artery (CRA) occlusion of the left eye” and was administered an intravenous infusion of dexamethasone and oxygen inhalation. She was then transferred to a second hospital, where she underwent interventional thrombolysis twice, but no significant improvement was observed. Cranial magnetic resonance imaging (MRI) revealed a few ischemic foci in both the frontal and left parietal lobes. The patient subsequently presented to our hospital for further diagnosis and treatment.

**Figure 1.**
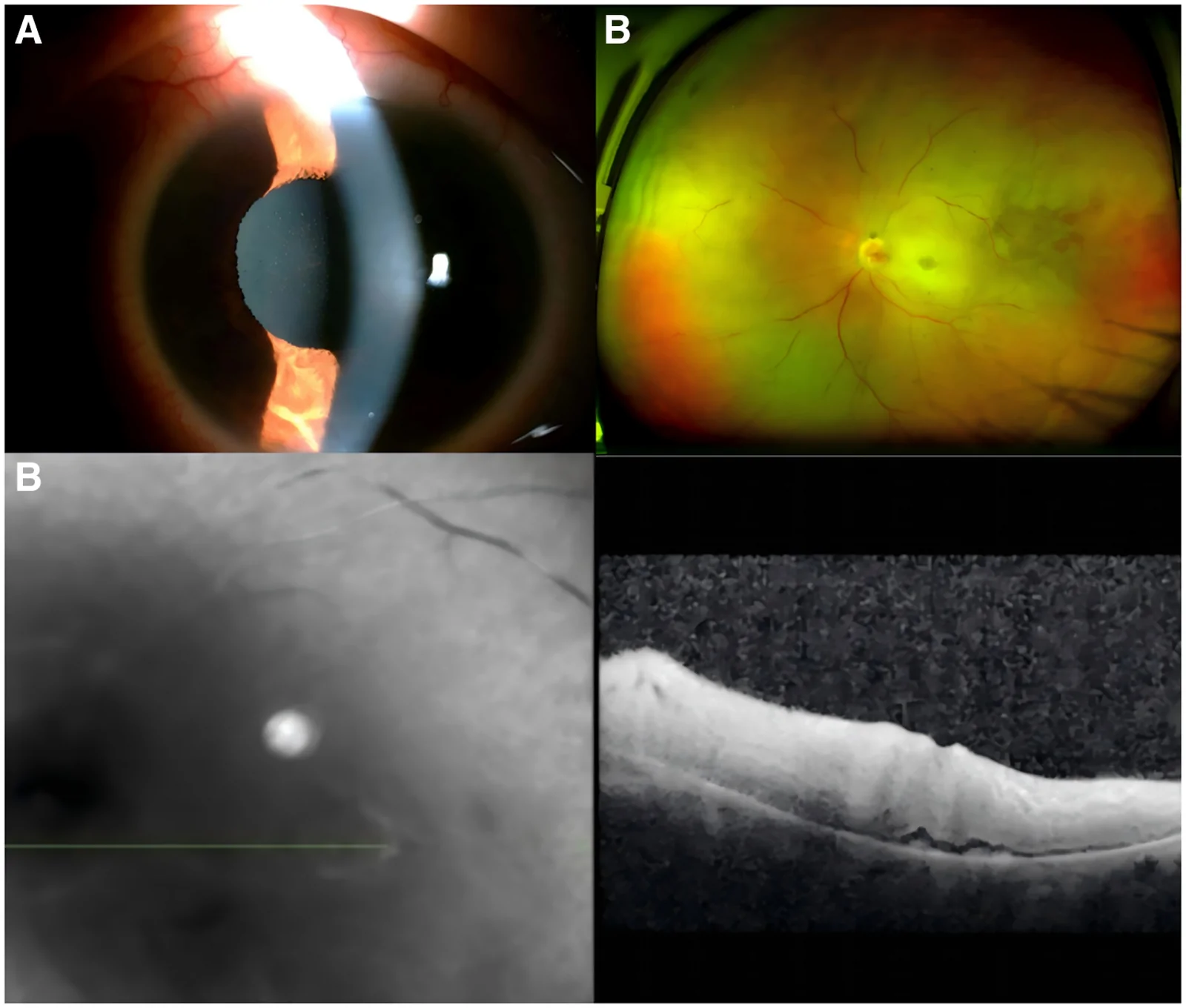
Anterior segment photography shows indistinct iris texture, mild pigment dispersion, a round and dilated pupil, and slight opacification with pigment deposition visible on the anterior surface (A). SLO A small reddish retinal patch was found in the superotemporal area of the macula, whereas the rest of the retina showed grayish-white edema with macular edema (B). OCT demonstrated diffuse retinal edema and thickening (C). OCT = optical coherence tomography, SLO = scanning laser ophthalmoscopy.

Physical examination revealed patchy changes in the skin over the nasal root and the left frontal region. Ophthalmic examination revealed an uncorrected visual acuity of 20/33 in the right eye, which could be corrected to 20/20 with −1.25/−0.50 × 22, and no light perception (NLP) in the left eye. The intraocular pressure (IOP) in the right and left eyes was 15 and 8 mm Hg, respectively. Slit-lamp examination revealed no obvious abnormalities in the right eye. In the left eye, no eyelid swelling, mild conjunctival hyperemia, clear cornea, mid-deep anterior chamber, aqueous flare (++), or inflammatory cells (++) were observed. The iris texture of the left eye was unclear, with a small amount of pigment loss. The pupil was round and dilated (approximately 6 mm in diameter), and the direct light reflex was absent. The lens showed slight opacification with pigment deposition visible on the anterior surface (Fig. 1A). Patchy white opacities were observed in the vitreous cavity. Funduscopic examination revealed an edematous optic disc with blurred margins and a pale white hue. The retinal arteries and veins displayed partial white linear changes with attenuated vessels, and the retina remained in situ. A small reddish retinal patch was found in the superotemporal area of the macula, whereas the rest of the retina showed grayish-white edema with macular edema. No cherry-red spots were detected (Fig. 1B).

As for auxiliary examinations, optical coherence tomography (OCT) demonstrated diffuse retinal edema and thickening (Fig. 1C). The P100 wave amplitude in the left eye was absent on flash visual evoked potential. A B-scan confirmed that the retina was in place. No waveforms were elicited in the left eye on the electroretinography. Based on ocular examination, B-scan, scanning laser ophthalmoscopy, flash visual evoked potential, electroretinography, auxiliary examination by OCT, and cranial MRI, the patient was diagnosed with left eye OAO.

The patient received integrated traditional Chinese and Western medicine treatment at our hospital. Upon admission, supportive anti-ischemic therapy was initiated immediately to improve retinal perfusion and provide neuroprotection. The treatment regimen included sublingual nitroglycerin, oxygen therapy, oral Compound Xueshuantong Capsules, intravenous inosine injection, intravenous Ginkgo biloba extract injection, intramuscular Mouse Nerve Growth Factor for Injection, and perivascular injection of Compound Anisodine Hydrobromide Injection adjacent to the superficial temporal artery. In addition, the patient received traditional Chinese medicine interventions, including oral Chinese herbal decoction and acupuncture. The herbal prescription was formulated according to the traditional Chinese medicine therapeutic principles of improving circulation and nourishing nerves, while acupuncture was administered with the aim of regulating qi, activating blood, resolving stasis, and unblocking collaterals according to traditional Chinese medicine theory.

**Figure 2.**
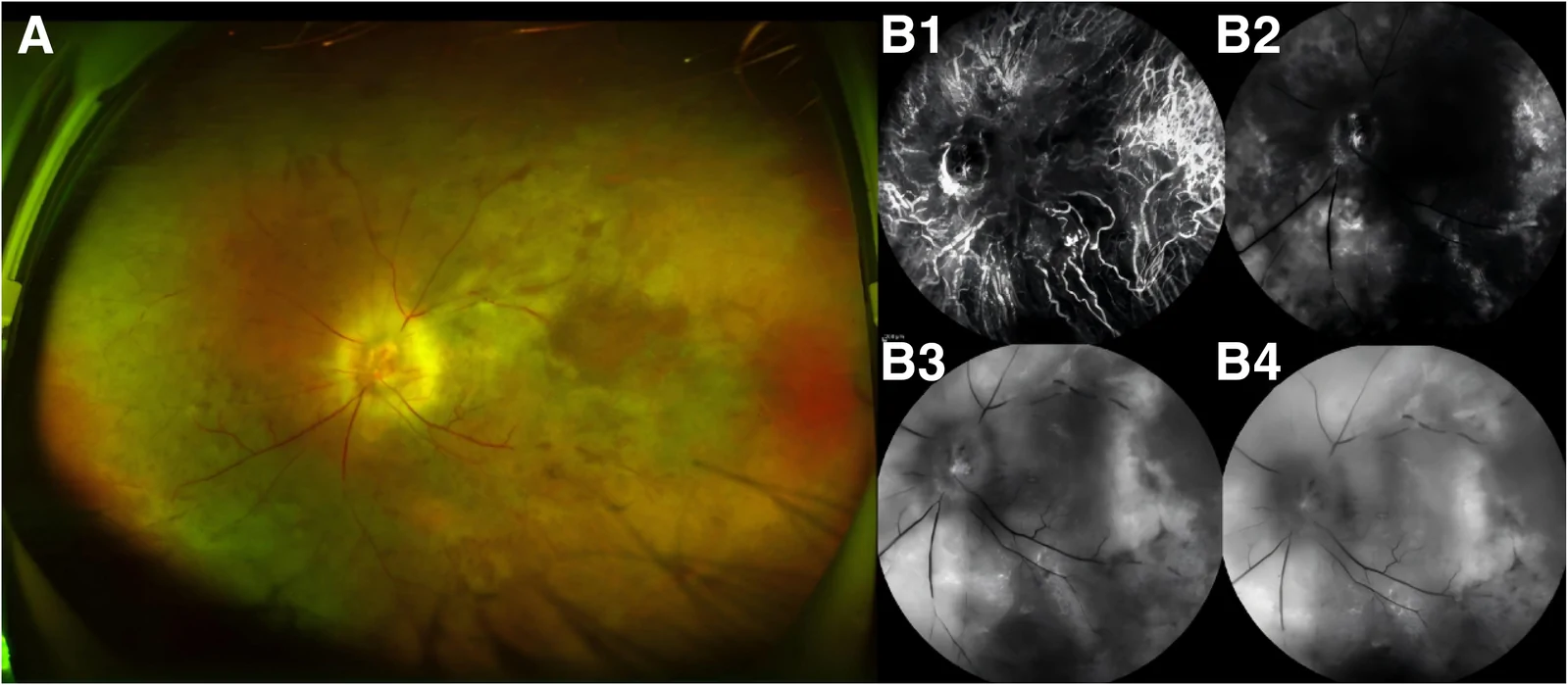
SLO revealed reduced optic disc edema, alleviated retinal edema, and reduced pigment deposition in the peripheral retina (A). ICGA showed only large choroidal vessel filling at 54 seconds (B1). FFA showed no retinal blood vessel filling (B2-B4). FFA = fundus fluorescein angiography, ICGA = indocyanine green angiography, SLO = scanning laser ophthalmoscopy

**Figure 3.**
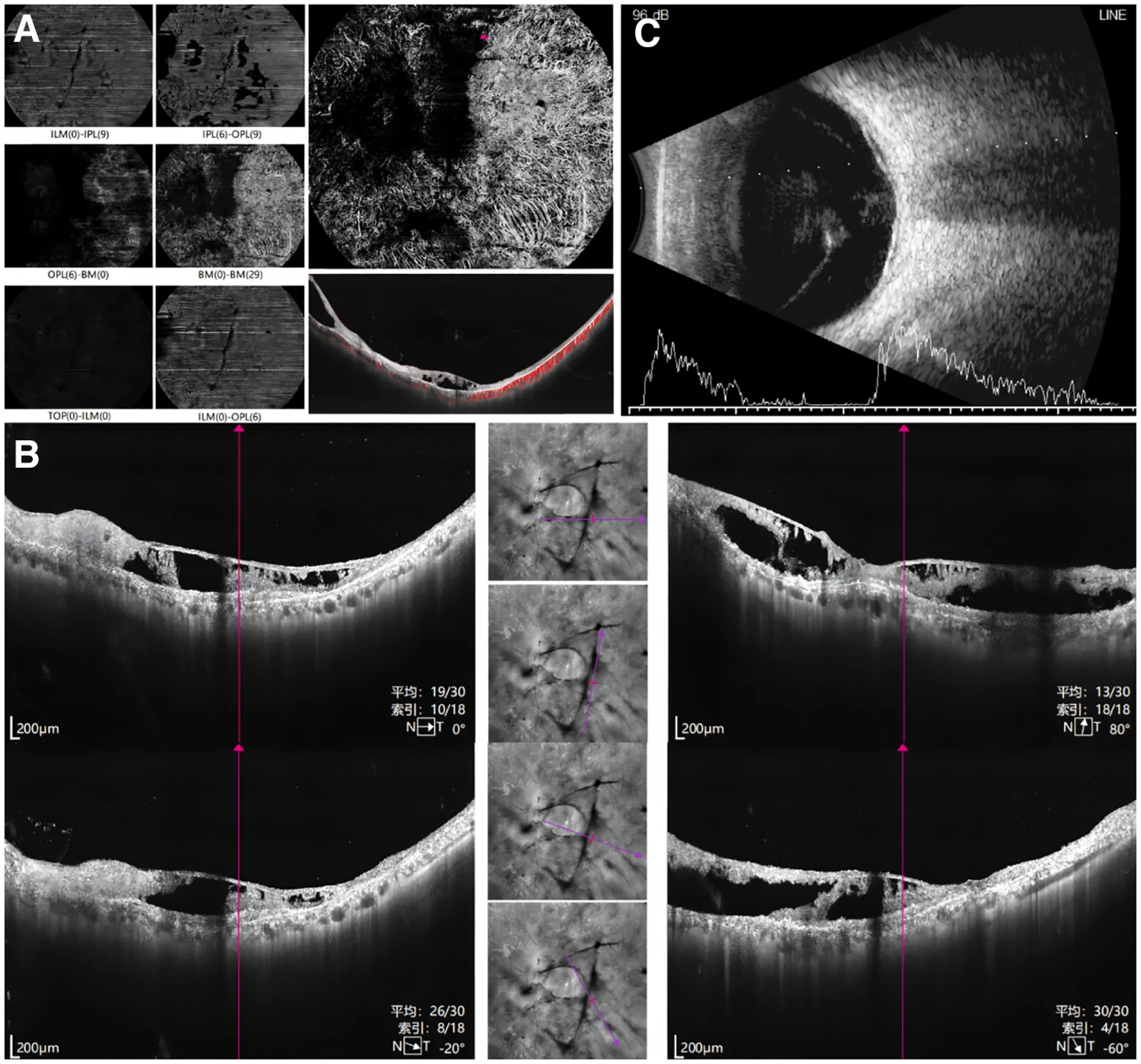
OCTA demonstrates no blood flow signals in any layer of the retina or the choroidal capillary layer (A). OCT multiple interlaminar splits in the retina and atrophic thinning of the peripheral retina (B). A B-scan showed retinal interlaminar splitting (C). OCT = optical coherence tomography, OCTA = optical coherence tomography angiography

At the 2-week follow-up, the visual acuity of the left eye remained NLP. Funduscopic examination revealed reduced optic disc edema, alleviated retinal edema, and reduced pigment deposition in the peripheral retina (Fig. 2A). Fundus fluorescein angiography combined with indocyanine green angiography showed no retinal blood vessel filling, and only large choroidal vessel filling was observed at 54 seconds (Fig. 2B). Optical coherence tomography angiography showed no blood flow signals in any layer of the retina or the choroidal capillary layer (Fig. 3A). OCT revealed multiple interlaminar splits in the retina and atrophic thinning of the peripheral retina (Fig. 3B). A B-scan showed retinal interlaminar splitting (Fig. 3C).

**Figure 4.**
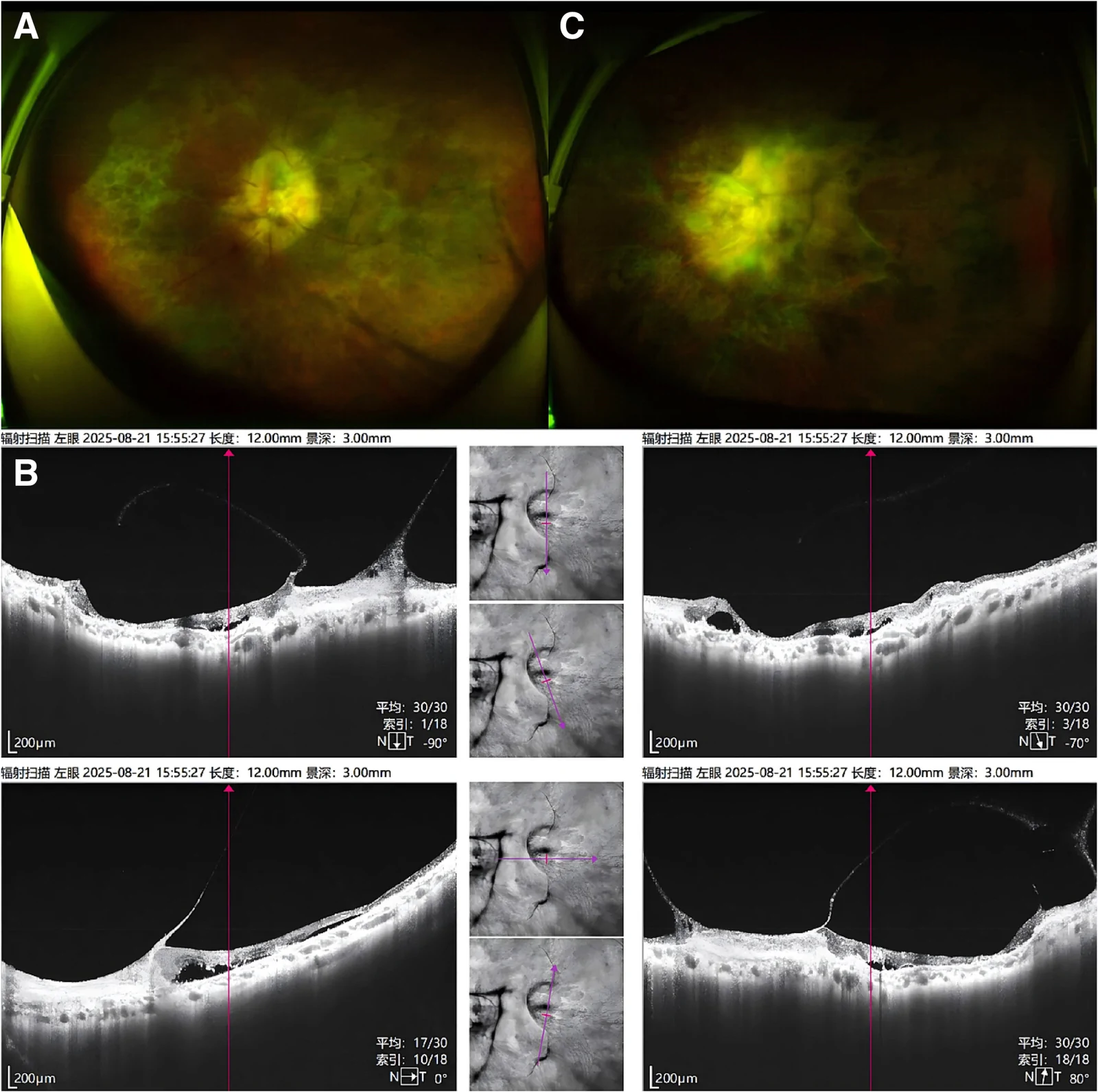
SLO shows an edematous optic disc with ill-defined margins and pale color, partial white linear changes of retinal arteries and veins, peripheral vascular occlusion, attached retina, diffuse grayish-white edema of the entire retina, macular edema, and an absent foveal reflex (A). OCT revealed retinal atrophy, disorganization of retinal structure, and partial traction from an epiretinal membrane (B). SLO showed a grayish-white epiretinal membrane on and around the optic disc, disorganized retinal structure, and patchy pigment deposition in the peripheral retina (C). OCT = optical coherence tomography, SLO = scanning laser ophthalmoscopy.

At the 4-week follow-up, the visual acuity of the left eye remained NLP, and the IOP was 12.7 mm Hg. Funduscopic examination showed an edematous optic disc with blurred margins and a pale white appearance, partial white linear changes in the arteries and veins, peripheral vascular occlusion, retina in place, diffuse grayish-white edema of the entire retina, macular edema, and no foveal reflex (Fig. 4A). At the 7-month follow-up, the visual acuity of the left eye remained NLP, and the IOP was 11.7 mm Hg. OCT revealed retinal atrophy, disorganization of retinal structure, and partial traction from an epiretinal membrane (Fig. 4B).At the 9-month follow-up, scanning laser ophthalmoscopy showed a grayish-white epiretinal membrane on and around the optic disc, disorganized retinal structure, and patchy pigment deposition in the peripheral retina (Fig. 4C).

## 3. Discussion

With the rapid development of minimally invasive aesthetic medicine, injectable cosmetic treatments have become the preferred option for individuals seeking beauty because of their convenience and immediate effects. However, despite the misconception of “risk-free aesthetic improvement,” cases of blindness caused by aesthetic injections continue to emerge. OAO is one of the most serious complications of facial filler injection.^[1]^ It severely threatens the vision of patients and causes irreversible eye damage and blindness. Therefore, further investigation of its pathological mechanisms, clinical features, and treatment strategies is essential. These studies can help effectively prevent this complication and provide timely and reasonable interventions to preserve patients’ vision.

The periorbital area has a dense vascular network; therefore, it is a high-risk area for visual injury caused by facial filler injections. The dorsal nasal artery is a terminal branch of the ophthalmic artery (OA). It anastomoses with the zygomatic, temporal, and lateral nasal branches of the facial artery through the angular artery of the medial canthal area. According to anatomical studies, the diameter of the angular artery is about 0.6 to 1 mm.^[7]^ HA, which is commonly used for facial filling, has different particle diameters owing to different product types and cross-linking degrees. The particle diameter of some products can reach 400 μm. Although this size is smaller than the diameter of the angular artery, fillers can still enter the CRA anterogradely or retrogradely through periorbital vessels, such as the angular artery, if the injection is performed improperly (e.g., excessive injection pressure). This can lead to occlusion of the CRA and further visual impairment. When the injection pressure exceeds the diastolic blood pressure of the arteries at the injection site (such as the dorsal, nasal, and angular arteries), the filler enters the main trunk of the OA along these arteries and forms an embolism. When the injection was stopped and the pressure decreased, the filler entered the branch vessels of the OA. If the injection pressure exceeds the diastolic blood pressure of the OA, the emboli can retrogradely reach the internal carotid artery and move forward to occlude the middle cerebral artery and anterior cerebral artery. This is a widely accepted retrograde embolism theory.^[2-4]^

Vascular depth also significantly affects risk. The central and paracentral arteries in the glabellar area are located at a mean depth of only 2.7 to 3.0 mm in the subcutaneous plane; therefore, they are easily punctured by mistake. These are branches of the OA. If fillers enter the OA retrogradely, the CRA may be further occluded. Autologous fat tends to form emboli in proximal vessels (such as the main trunk of the OA) because of its large particle size and high viscosity, while HA may occlude distal branches.^[8]^ In 60 cases of visual injury caused by HA fillers, Kapoor et al found that nasal injections accounted for 56.3%, glabellar injections for 27.1%, and frontal injections for 18.8%. These areas are the main high-risk zones.^[9]^

In this patient, the injection site was located at the nasal root, where the dorsal nasal artery runs. Acute visual loss in the left eye occurred during HA injection into the nasal root, and obvious signs and symptoms of ocular ischemia were observed.

OAO presents as reduced blood supply to the retina and choroid, with typical clinical manifestations. First, patients usually experience sudden, painless, and severe visual loss, mostly without light perception, and there is usually no obvious improvement in vision after treatment. Patients often present with ischemic optic neuropathy and afferent pupillary defects. Fundus examination mainly shows diffuse edema of the whole retina, usually without a macular “cherry red spot.” In addition, slit-lamp examination mostly shows noninfectious inflammation of the anterior segment, such as corneal edema, anterior chamber flare, and pupillary dilation > 5 mm. Most patients experience ocular pain, ophthalmoplegia, or strabismus. Cranial MRI usually reveals focal or diffuse cerebral infarction.^[10]^ Patients with filler-related ischemia often have skin lesions in the areas of arterial occlusion that show pallor, mottling, or necrosis.

In this patient, ischemic foci were found bilaterally in the frontal and left parietal lobes before admission. After admission, physical examination revealed mottled skin changes at the nasal root, visual acuity with NLP, IOP of 8 mm Hg, and significant noninfectious inflammation of the anterior segment. These findings are consistent with the aforementioned typical features.

Currently, there are no established guidelines for the treatment of iatrogenic OAO. Standard rescue treatments for OAO include IOP reduction (e.g., ocular massage under fundus monitoring, anterior chamber paracentesis, and oral methazolamide), anticoagulant therapy, glucocorticoid application, and hyperbaric oxygen therapy.^[5,11]^ These treatments can reduce tissue edema and inflammation, improve tissue hypoxia, enhance the survival of endangered visual cells, and improve vision. These interventions are typically used as basic treatments in combination with arteriovenous thrombolysis.^[12–14]^ Arteriovenous thrombolysis is considered the most direct method of treating injection-related OAO.^[13,14]^ Compared to conventional thromboembolism, combined thrombolytic injection of tissue plasminogen activator and hyaluronidase may have a better thrombolytic effect than hyaluronidase alone in the treatment of HA-induced OAO.^[1,15,16]^However, all these treatments require high-level evidence-based medical support.

With current treatments for OAO, the overall visual recovery rate of this disease is <20% (30/146 cases).^[6,17]^ Incomplete occlusion has a significantly better prognosis than complete OAO because part of the retinal perfusion is preserved. Many studies have shown that if treatment is received within 4.5 hours of symptom onset, the visual recovery rate is close to 50%.^[6,18]^

Previous reviews have suggested that ophthalmoplegia may improve over time, whereas pupillary abnormalities often persist and appear to have a poorer prognosis.^[19]^ This is mainly owing to the ischemia of the ciliary ganglion, which gives rise to postganglionic fibers that innervate the intraocular muscles. Its mitochondrial vacuolation rate is high, and ATP synthesis is interrupted, leading to calcium overload and selective necrosis of postganglionic unmyelinated fibers.^[20]^ In this case, the patient missed the optimal rescue window, and systemic treatment was initiated after embolization. Ischemic necrosis of the retina was observed, and visual recovery was impossible.

Blindness caused by aesthetic facial injections results in severe visual damage that is difficult to treat clinically. Currently, there are no effective rescue measures; therefore, prevention is of the utmost importance. For specific prevention, first, operators should fully understand the anatomy, location, and depth of facial blood vessels and choose to inject directly into the supraperiosteal plane or into the superficial dermis. These areas are safer than deep subcutaneous injections. Second, the operation should be performed slowly and gently, injection pressure should be strictly controlled at a low level (<100 mm Hg), and the needle should be moved slowly during injection. Third, instrument selection is very important. It is recommended to use cannulas of an appropriate size (e.g., 25G and larger, as recommended by studies). The risk of arterial wall penetration is significantly higher with 27G and smaller cannulas.^[21]^ Finally, targeted digital compression can be used in clinical practice to block major periorbital blood vessels and prevent accidental retrograde flow of fillers.^[22]^ A cadaver study also confirmed that digital compression at the nasal root during cosmetic filler injection, particularly in high-risk areas such as the nose, can effectively reduce the risk of fillers entering the orbit.^[21]^

As filler injection technology is widely used, practitioners should implement systematic prevention measures across multiple perspectives, such as strengthening risk awareness, improving professional skills, standardizing operational procedures, and optimizing emergency plans. These measures can reduce the risk of blindness caused by filler injections and effectively protect patients’ medical safety.

## Author contributions

**Conceptualization:** Ziqi Xiong, Yane Gao.

**Data curation:** Ziqi Xiong, Haiming Lv.

**Formal analysis:** Ziqi Xiong, Haiming Lv, Chunhuan Niu, Ying Wen.

**Investigation:** Haiming Lv.

**Supervision:** Yane Gao.

**Writing – original draft:** Ziqi Xiong.

**Writing – review & editing:** Haiming Lv, Chunhuan Niu, Ying Wen, Yane Gao.
